# “Making it okay”: professionals in high-stress environments construct their understanding of the impact of a yoga-based retreat designed to build resilience

**DOI:** 10.1080/17482631.2022.2025640

**Published:** 2022-02-14

**Authors:** Tegan J. Reeves, Natalie L. Dyer, Sara Borden, Jeffery A. Dusek, Sat Bir Singh Khalsa

**Affiliations:** a Global Pediatric Medicine Culture & Communication, St Jude Children’s Research Hospital, Memphis, TN, USA; b Global Pediatric Medicine Culture & Communication, Kripalu Center for Yoga & Health, Stockbridge, MA, USA; c Global Pediatric Medicine Culture & Communication, Brigham and Women’s Hospital, Harvard Medical School, Boston, MA, USA

**Keywords:** Yoga, qualitative, resilience, stress, awareness, integration, engagement, mindfulness

## Abstract

**Purpose:**

While there is growing evidence for resilience building programmes, to date research has not explored how professionals construct understanding of programme impact. RISE (resilience, integration, self-awareness, engagement), a 5-day yoga-based retreat programme, has been linked with positive wellness outcomes. This qualitative inquiry explores participants' reflection and experience 3 months after programme completion.

**Methods:**

Through a grounded constructivist lens, in-depth semi-structured phone interviews were conducted with 17 adult professionals in high-stress work environments who attended RISE. Initial hand-coding informed codebook development for systematic coding using directed content analysis using sensitizing structuring.

**Results:**

Two integrated perceptions woven through five themes. Persistent threads of experiential learning and sense of permission provided structure for themes found. Five interrelated themes related to psychological health and workplace dynamics were (1) use of acquired behavioural skills and practices; (2) lived mindfulness; (3) resilience to stress and emotion regulation (4) self-care and self-compassion, and (5) sharing with others.

**Conclusion:**

Findings provide meaningful interpretation of previously reported programme efficacy by contextualizing perceived benefits within participants' constructed understanding of change. Specifically, environmental, social, and experiential considerations have suggested implications for resilience building programmes.

**Abbreviations:** RISE (resilience, integration, self-awareness, engagement)

## Introduction

Professionals who work closely with at-risk, vulnerable, or dangerous populations in a specialized area such as education, health care, or social services, and are exposed to high stress scenarios and greater risk for developing burnout, disease, and mortality (Eddy et al., 2016; Ganster & Rosen, 2013; Ray et al., 2013) Systematic reviews of yoga-based interventions report that yoga decreases stress and burnout, and increases coping, self-care and resiliency thereby promoting health, functionality and wellbeing (Domingues, 2018; Ofei-Dodoo et al., 2020). Yoga decreases anxiety in depression with clinical populations (Kwok et al., 2019). Over several years, Kripalu Yoga programmes have demonstrably improved psychological and physical health indicators in many populations, including youth in schools (Butzer et al., 2017), police officers (Jeter et al., 2013), obese populations (Braun et al., 2012), mental health professionals (Riley et al., 2017), educators (Trent et al., 2019a), physicians (Scheid et al., 2020), and heterogeneous groups of frontline professionals (Dyer et al., 2020; Trent et al., 2018, 2019b).

The yoga-based secular programme called RISE (resilience, integration, self-awareness, engagement), was developed for and offered to frontline professionals. Help at Kripalu Center for Yoga and Health, the programme improves psychological health and wellbeing in frontline professionals, including mindfulness, resilience, empowerment, self-compassion, and affect, with most improvements persisting up to 2 months following the programme (Trent et al., 2018, 2019). Studies with longer term follow-up show that improvements persisted up to 6-months following RISE (Dyer et al., 2020). While these quantitative results show encouraging support for the RISE programme, experiential perceptions of the benefits and memories of the programme have not been explored. In order to enable consideration within a wider context, inquiry should pursue a more robust understanding of why and how participation influences change (Denzin & Lincoln, 2005). In addition, qualitative exploration may also shed light on individual differences that may influence programme feasibility and also provide valuable data that can inform programme improvement. This is the first qualitative investigation of the RISE programme. The current study has two aims: (1) to explore in-depth recollection of participants’ RISE experience; and (2) to explore participant reflections on of the perceived impact RISE long term (3-months post-programme).

## Materials and methods

To pursue the aims of the study qualitative methodologies were employed through a grounded constructivist lens. Specifically, pursuant to the first aim, a pragmatic epistemic framework of inquiry was used to elicit in-depth recollections of experience. Pursuant to the second aim, constructivist theory was used to explore perceptions of applied resilience. Both pragmatic epistemology and constructivist theorists view knowledge as constructed from experience. Thus, understanding participants’ perceptions were elicited from interpersonal participatory communication about experience (Rubin & Rubin, 2005) In-depth interactive interviews were conducted to collect rich and descriptive data from participants (Patton, 1980). Using constructivist grounded theory, a collective construction of understanding of perceived behaviour change as a result of participation was developed through pattern and content analysis (Hsieh & Shannon, 2005). In addition to rich and descriptive data, a positivist frame was used to pursue the second aim of the qualitative study: to explore the perceived impact of participation in the 5-day residential RISE immersion programme (Charmaz, 2006). In this vein, qualitative materials were developed to elicit both fact-producing interaction (Gomm, 2004) and experience exploration (Rubin & Rubin, 2007).^17^

### Resilience program

RISE curriculum designed was designed specifically to address the challenges faced by professionals in positions with innate stress and trauma exposure. Attendees arrived at Kripalu, in Stockbridge, Massachusetts, where they slept, dinned, and attended five hours of structured sessions for five consecutive days. During their free time participants were able to take yoga classes, hike, and commune on the grounds of a scenic centre overlooking the Berkshire Mountains. In many cases, participants stayed in rooms with other guests and shared bathroom access. RISE was developed as a comprehensive programme of yoga-based practices, skills, and techniques including yoga postures, breath regulation, relaxation techniques, a variety of meditation formats (loving kindness, concentrative, mindfulness), body scan, mindful communication, and a focus on healthy behaviours concerning physical activity, nutrition, and sleep. Curriculum elements included yoga sequences, sitting meditation, breathing practices, and education sessions. Educational material included mindful communication didactic exercises; sleep information and preparation for mindful wind-down; and eating in mindful healthy ways. The RISE programme is described in extensive detail in the quantitative publication (Trent et al., 2018).

### Participants and process

Frontline professionals were invited by Kripalu through their partnerships with local organizations, to attend RISE. All attendees were invited to take part in a qualitative interview. Self-selection sampling was chosen to provide meaningful conversation about their RISE experience, as such this is both a purposive and convenience sample. Interviewees were given a $15 Amazon gift card upon completion of the interview.

RISE interview participants were recruited via email approximately 3 months after programme attendance. Participants who expressed interest were contacted by the interviewer. Interviewers, including the first author, were trained in interview techniques and methodology. Both interviewers were female with familiarity with, and experience at, Kripalu. To elicit authentic reflection, phone-interviews were employed. This allowed participants to feel comfortable in their home environment after having re-engaged with day-to-day life and reflect on the impact of the programme from a perspective outside of the retreat centre.

Interviews were participant led (Clough & Nutbrown, 2002) and inquiry-based conversation (Castillo-Montoya, 2016). Semi-structured interview guides (see appendix) were developed to elicit conversational narratives of participants’ perceived impact of the RISE programme after time. Interviewers introduced themselves as research team members from Kripalu. With participants’ permission, qualitative interviews were audio-recorded and were up to 50 minutes long.

The interviewer (TR) specializes in qualitative inquiry/incorporation and has extensive experience with in-depth interviews as well as RISE programme material. To emphasize a shared perspective and allow for reflexive and in-depth inquiry, the interviewees were made aware of the interviewers’ position both as an independent researcher and someone who had attended Kripalu yoga-based programmes.

Of the selected cohort of 64, RISE programme participants were invited to participate in the interview, 17 programme attendees agreed to and completed the interview (26.6% of attendees). Participants (n = 17) were 94.1% female and 5.9% male with a mean age of 40.3 years (25–65). Interview participants represented a similar sample of professions as RISE participants including social service workers, first responders, and inner-city youth programme directors.

### Data analysis

Phone-interviews were transcribed verbatim and coded using initial hand coding and secondary NVivo qualitative analysis software, version 10 (Castleberry & Nolen, 2018). Using directed content analysis (Hsieh & Shannon, 2005) and sensitizing structure (Charmaz, 2006), interview transcripts were inductively coded for initial patterns via random selection of 5 interview transcripts. While basic epistemic foundation of a grounded, experiential, and in-depth approach to the exploration at the inception of the study, coders collaboratively reviewed methodologies and decided on constructivist scaffolding to analyse the data. After initial coding, two independent coders reviewed discrepancies, converged ideas, and discussed saturation and/or singular importance. Upon consensus, descriptive codes were presented to additional members of the research team for review and approval. Using this preliminary codebook of minor themes, all interview transcripts were then coded using a constant comparative approach (Glaser & Strauss, 1967) to elicit persistent perceptions (or minor themes). To strengthen the trustworthiness of the iterative coding process, consensus meetings and discussions throughout coding led to identification of categories (or major themes). Exemplar statements and narrative construction of themes are provided.

## Results

Pursuant to the first aim of the current work five main categories were elucidated: (1) use of acquired behavioural skills and practices; (2) lived mindfulness; (3) resilience to stress and emotion regulation; (4) self-care and self-compassion; and (5) sharing with others. These themes are explored in detail below.

Pursuant to the second aim, participants’ reflections of their experience were reviewed for valance. Accounts of experience at RISE were positive, commonly citing the retreat experience as transformational. Participants offered layered narratives of learning and growth. For example, one participant described the retreat as reflective time for somatic awareness immediately linking it to motor ability. Inferring that RISE impacted physical and somatic wellness she said: “the quiet walks, the quiet breakfasts, you know, learning how to feel that energy within my body. From the day I got there I had to sit on the chair to when I left I could actually to sit down on the floor with everyone.” (Female, 51) While common themes are detailed further, it should be noted that the experiential nature of the data. Participants referenced an integrative experience of wellness learning, often citing food and lodging. For example, “but to actually experience it and learn about different spices, and to you know just to see like, ‘Oh wow, I can get this in my community, I’ve seen it, I didn’t know what to do with it. I didn't know what it tastes like,’ you know, to have that exposure, made a tremendous amount of difference, and then the experience of the quiet breakfast, you know” (Female, 51). In addition, evidence of the wellness outcomes of the curriculum is displayed by reported use of explicit teachings.

Themes of *use of acquired behavioural skills and practice, lived mindfulness*, and *resilience to stress and emotion regulation* support the explicit impact of the RISE program. Participants shared changes in behaviours perceived to be a result of the program. Specifically, *self-care and self-compassion* as well as *sharing with others* the practices, constructs and experiences with others were reported across interviews. Table I shows the themes derived, definitions, and an example of each.

**Table I.** Codebook and theme development

Initial descriptive codes	Persistent perceptions within descriptive code	Themes derived	Definition	Example
Tools	Inner tool Specific use of tool Connection to well-being	Acquired behavioural skills and practices	Continued use of inner resources/tools to promote well-being.	*So, I at least do, like, you know, loosen up my shoulders, and I’m thinking about it, and I’m thinking about breathing.*
Mindfulness	Experiential learning Experiential awareness	Lived mindfulness	Awareness of the mind and body and a practice of that present awareness.	*I feel like I’ve like using mindfulness, and being present, I’ve been able to harness the joy from some of the things that I might not be paying attention to.*
Coping	Resilience to stress	Emotion Regulation	Increased resilience, particularly related to decreased levels of stress.	*I think I have remained calmer, you know,, through some really difficult situations*. *So, my level of anxiety has gone down like you would not believe*. *It’s like that’s the biggest thing that I’ve noticed, and I just don’t stress out about things like I’m used to.*
Self-care	Allowing Boundaries Self-acceptance	Self-care and self-compassion	Allowing oneself to engage in behaviours that are beneficial and kind to the self.	*… trying to find a balance of also taking care of myself and doing activities that are rejuvenating for me as a caregiver …. I’ve seen changes in my co-worker that went to the facility. I feel like he’s taking better care of himself now. And he seems to be in a really good place.*
	Teaching tools Creating Culture Sharing	Sharing	Participants’ sharing and teaching the tools learned through the programme to others.	*The breathing, reminding them that we will take breaks and that they can take a break on their own if it gets intense. And also, we remind each other to leave stuff outside, what we call “outside the door” so that we can be fully present during the time. The other thing, I think is, I tend, when I do groups, to brings snacks, and I’m going to be a lot more mindful about the kinds of snacks that I bring.*

Collaboration between researchers was used to develop consensus on themes. Inquiry audit was used to ensure dependability.

### Use of acquired behavioural skills and practices

Reflection on the RISE programme offered varying usable tools and resources replicated after programme completion. Participants reported continued use of inner resources/tools to promote their own wellbeing, commonly citing the tools as a formative effect of the programme. For example, while stating the impact of the programme one participant described: “Honestly the tools, like the breath, the things that focus and get us back to the present has been the most useful, because once I’m focused and present in my situation and body, I can see the future in a more doable way” (Female, 31). Many participants described situations that suggest transformation through use of tools that were learned in the programme. For example, one participant discussed improved stress reaction, or emotion regulation, through using breathing tools:

I [was] very quick to react. But now I’m learning how to breathe in response. … are some situations that I have to react to, but taking that step, you know, breathing, holding a belly breath, releasing it to the top of my lungs. You know, it’s completely, [a] completely different outcome. (Male, 34)

**Figure 1.**
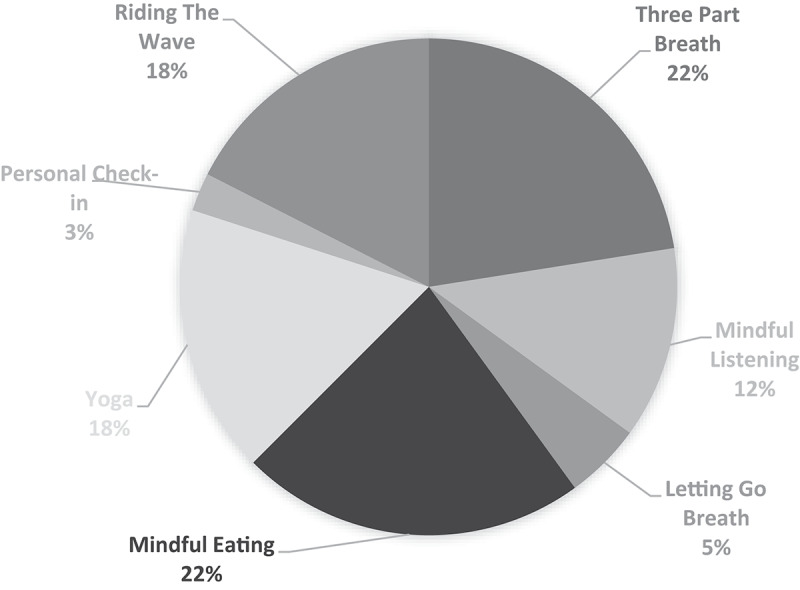
Frequency of tool mentions. Frequency counts based on number of times mentioned: no weighting method used.

It should be noted that these expressions of acquired behavioural skills and practice are directly related to specific content in the curricula. Therefore, these expressions should not be perceived as naturally emerging from the experience rather reflective of the tools that first came to participants' minds 3 months after the retreat. As tools were embedded in the RISE curriculum, frequency counts were used to evaluate the scope and weight of explicitly taught items. Table II shows the tools mentioned by participants with an example of each. Figure 1 shows the frequency of the tools being mentioned.

**Table II.** Perceived tools and examples

Tool	Example
Complete Breath	“ … amazing to learn the three-part breath”
Mindful Eating	“ … mindful eating … I practice a lot since … ”
Riding the Wave	“ … riding the wave works the best for me”
Postures and Physical Exercises	“I have been practicing yoga since, since that retreat”
Mindful Listening	“ … mindful listening is definitely helpful”
Letting Go Breath	“The letting-go breath. I use that all the time”
Personal Check-in	“ … the personal check-in that they taught us”

Tools used were based on interview coding and not collected from RISE Curriculum however researchers had implicit understanding of the curriculum and tools to ensure expert evaluation.

### Lived mindfulness

Participants described mindfulness within experiences of moments. Mindfulness in day-to-day life was described as experiencing, rather than performing, tools of objective awareness of both mind and body. This distinction of lived mindfulness emerged as a persistent component distinguished from typical mindfulness discussion. Accounts of mindfulness were made as descriptive skill use, related to reports of transformation, empowerment, and/or improved communication. For example, one participant states “ … becoming aware of my breath makes me able to become more present and aware of like my body and what the actual reality is as opposed to. Like you know. Whatever fears and things like that are just being generated from a busy mind.” (Female, 31). This integrated account describes an experience of mindfulness as well as a demonstration/performance of mindfulness.

The transformative quality of lived mindfulness was often discussed. For example, “… for the first time I realize how much I am running and going from thing to thing without really thinking about what I’m doing” (Female, 53) and “I really feel like my life has changed a lot. You know, from small little things, like when I go and take a walk, you know, noticing things, the sounds, and the smells, and the sensations, which I never really did before.” (Female, 49). Others elaborated on a sense of expressed empowerment as one participant illustrated:

My [habit of] not being present was a way of avoiding things. So, I think like it’s empowering to imagine that like “Oh, that I can actually be here while this unpleasant thing is happening, and I can survive it, and I can-” You know, it gives you a sense of accomplishment to be able to deal with something and actually deal with it. (Female, 31)

These frontline professionals expressed poignant transformation related to their improved ability to communicate in seemingly stressful interactions. For example, one participant perceived her own mindfulness as influencing the social sphere around her saying “ … It's kinda like an instant reset when I am mindful and focused and present in that way, the other person kinda responds by settling down also.” (Female, 59). Participants shared that as behavioural health professionals this helped them in leadership and communication, as one participant explains:

I’m able to slow myself down and be completely present to the person I’m supervising, and I deliberately do that instead of kinda feeling all of my responsibilities, I’m able to just narrow down my focus to the person who I am talking to and just doing that consciously makes a huge difference first of all in how I feel about the conversation and also the effectiveness of the conversation. (Female, 59)

### Resilience to stress and emotion regulation

Further expressions of transformation were reported through participant reports of increased resilience. In particular, resilience was connected to ability to cope because of decreased levels of stress. Specifically, participants suggested an ability to recover quickly during challenging moments. There was a range of situated stress from small stressors as one participant describes; “Things don't bother me as much. Like, even if I’m running 5 minutes late, it’s, you know, taking a few breaths and saying, like you know, “A week from now, it’s not going to be a big deal that I was 5 minutes late” (Female, 27), to potentially traumatic stressors as another participant describes; “When I realized [a gunshot I heard] was close to me physically, it was three blocks away. I … my first response was to open that [mindfulness] app and just take time to just, to just be quite and still and kind of give myself some comfort.” (Female, 53).

Similarly, participants described diverse mechanisms of resilience. One participant describes it as an *ability to let things go*: “I am better able to kinda let things, not let things go, but like I said deal with it and then move on. Instead of, you know, keeping on and stressing on about one specific thing. You know I let my feelings be known, and then we go from there” (Female, 49). Another participant discussed an ability to take on perceived stress (through social interactions) with less impact: “It's like easier for me to cope with a lot of interactions. It would get overwhelming if I spent like 5 days in a row around a lot of people. But it's not as bad anymore. Like, I can recharge even with other people around sort of” (Female, 25). While still others described resilience as a type of emotional regulation. For example, one participant said her anxiety was seemingly *gone* after the programme:

Before I went [to RISE], I had a lot of stuff going on in my life, and I was having a lot of anxiety. And it was affecting me, like I would have panic attacks and things like that sometimes. But when I went to the Immersion program, like it was gone. My level of anxiety has gone down like you would not believe. It’s like that’s the biggest thing that I’ve noticed, and I just don’t stress out about things like I used to. (Female 54)

### Self-care and self-compassion

A surprising element of the experiential and transformative benefits of RISE was the increased ability for self-acceptance and boundary setting. Participants reported engaging in behaviours that are beneficial and kind to themselves. Interlaced in reports of self-care was also an expression of self-*allowance*. Self-care seemed to arise from the interviews in a sense of permission to slow down, take breaks, and notice surroundings. “It's incredible how much it's shifted, and like I sleep in a little bit later, I get up, you know, I do, I sit and have breakfast, I walk my dog, and then just kinda go into work. Everything is just paced a little bit slower.” (Female, 27). These reports were in the context of self-awareness and self-acceptance. In respect to self-awareness a connection to noticing ones’ level of stress allowed for more self-care. For example, one participant describes burnout:

So, all of those other intrusive thoughts would come in which also signaled to me of being a little tired or burnt out or what-have-you. So, being able to, it’s almost like self-soothing, you know, where I can listen, if I like, ‘Ok, I recognize that I’m not, that I’m starting to not pay attention or heard the car or someone just came into the office, let me re-focus on what this person is saying.’ (Female, 51)

Indeed, this self-soothing tool seemed to have a lasting impact on participants. One participant elaborated on transformation in a sense of *willingness*. She states that allowing herself to take a break allowed her to readjust “I might otherwise have just plowed along gotten exhausted or something like that. So, taking care of me, adjust-, shifted a bit as a result of the [program].” (Female, 65). As the former RISE participant suggests, a normalized view of a responsibility to continue work even in the face of negative outcomes, as she puts it *ploughing through*, may be why so many participants feel that the first step in self-care was allowing or permitting oneself the action. Also considered a component of mindfulness, this form of self-acceptance was expressed in choices to take time other areas such as food intake.

Not to feel guilty about eating something that is not good for you and to just fully enjoy it. I think that makes it easier to make healthful choices because you don't feel like you are breaking the rules when you make a not as healthful choice. It was like a really, a really important shift for me in thinking of like you know really enjoying each experience for what it is without, without attaching a judgment to it. (Female, 25)

### Sharing with others

While intraindividual self-acceptance and self-compassion was often discussed, interindividual reports also arose from the interviews. A construct not explicitly taught through the programme that emerged as a perceived outcome was participants’ sharing with others the practices, constructs and experiences from the programme. For example, one participant increased in excitement when talking about the tool *letting go breath* and describing the effect being multiplied and saying, “I share them! I like to do it myself and I share with others.” (Female, 54).

As frontline professionals are often working with challenging populations, the use of teaching internal tools to others seemed to be a commonly referenced impact for participants. As one participant who works at a juvenile rehabilitation centre said, “… using those skills in our facility was also extremely helpful.” (Female, 31). Tools referenced varied but often included breathing and yoga techniques as one participant shared: “We do the like sun salutations as a way to end our session and get them to take some nice, letting-go breaths.” (Female, 54). The tools most commonly cited as something used in the frontline professional’s work setting to teach had to do with breathing. It seemed particularly useful in situations dealing with anxiety and stress. Once participant describes this:

I’ve had a lot of kids who are having like panic attacks and like just a lot of distress after being restrained. So, just kinda being with them and having them follow my breath to get them to focus and calm down and be able to talk has been helpful for them. (Female, 31)

Some participants described sharing with others the practices, constructs and experiences after the RISE programme such as the “experience within”. Often this was in the context of bringing presentations and education to outside groups. For example, one participant spoke about sharing the experience at work saying, “I have the experience within me and just try to communicate to other people, “Hey, I want to share this experience.” (Female, 51). Shared language was also used to describe educational and informational programming that the participants used to share their learning at RISE. For example, one participant described programmes created at a local school:

I participated in some parent education groups at a local elementary school, and we, that’s what we called it, “riding the wave of, riding the wave of parenting,” and then we identified 5 workshops based on some Kripalu language: “Staying calm when your child cannot,” “how to stay calm in a hectic world.” So, so, that really just solidified that even more for us as a, as a whole agency. (Female, 57)

An emergent concept from the participants was intentional sharing of practices and constructs with inference to a desired shift workplace culture. Participants shared ways in which they used their experience to cultivate a shared culture and environment of resilience-based pursuit. Through the interviews, this holistic integration of environment and tools was a common theme. Overlapping codes of transformation, tools, mindfulness, and sharing often painted a picture of a centred approach of resilience integration. Some participants demonstrated this through interlacing tools, experience, and relationships in multiple components of the workday. One participant described this holistic/integrated transformation as seen through co-workers who also attended the RISE programme:

The environment we work in is very almost militaristic and very like team-oriented, and it’s very much like, you know you don’t let other people down and sometimes self-care is seen as like selfishness if someone is like, “Oh, I need to take a break.“ But I see that people are making time for themselves. And I think it’s just because you know that individuals that went to Kripalu or you know are practicing mindfulness, and you know, self-care and things like that are kinda speckled in. So, I think it’s, it’s making it ok for people to say that, “I’ve had enough.”, “I’ve set good boundaries”. (Female, 31)

## Discussion

This paper presents participants’ reflections on usable skills, learning transfer, and transformative experiences of participation in the RISE programme. Of note was the shared construction of understanding through lived experience and a sense of permission. Permission for self-care and compassion was described as a novel lived experience by participants. For many, this feeling of “making it ok” (Female, 31) provided structure for expressions of their ability to cope with stressful situations and difficult conversations. In conjunction with the enthusiastic towards sharing their experience, this suggests a social and experiential component to the curriculum. In his book, *Permission to Feel*, Marc Bracket describes a similar expression in students learning to express emotional responses. While it is beyond the scope of the current paper, our data does suggest that perceived positive impact of the RISE program related to the ability to experience the learning process in an environment that felt safe.

The professionals who attended the RISE programme described using yoga-based practices to improve resilience. They further described experiences, concepts, skills, and practices that they believed led to improvements in psychological health and wellbeing. Participants also reported ways in which they shared yoga-based practices with others suggesting a belief that the skills learned could positively impact others and a desire for others to benefit. These findings give meaningful depth to the quantitative reports of empowerment and positive affect 2 months following participation (Trent et al., 2019b). Additionally, previously published quantitative reports from the RISE programme present evidence of improved mindfulness, resilience and self-compassion (Trent et al., 2018) which are corroborated by the current qualitative reports. Our current findings provide important context and add layered meaning to the potential impact of the RISE programme.

The current findings also add nuance to understanding the impact of yoga-based tools on frontline professionals. It is commonly accepted that mindfulness is defined as purposeful attention to any given moment without judgement (Kabat-Zinn, 2009). While this was clearly expressed by the participants, a more nuanced view of mindfulness, involving a *lived* experience of mindfulness components, adds a richer dimension to the current definition. Descriptions of mindfulness were embedded in day-to-day life stories in which “ability-to”; “habit-of”; “objective-observation-of”; and “reflection” were underlying changes in a response to surroundings. Higgins and Eden (2018) discuss a similar concept transformation through experiential awareness as a way of learning leading to a deeper understanding of one’s mindfulness. Similarly, frontline professionals expressed mindfulness as a lived experience, rather than a direct performance, which allowed their direct objective purposeful attention. As such, participants infer transformation through lived mindfulness.

In addition to the use of tools to support resilience, participants described a state of being more resilient. Examples of resilience implicitly included emotion regulation through the use of both intentional and observed-unintentional use of mindfulness and other tools. This resembles Braunstein’s framework outlining a layered and dynamic learning process of emotion regulation (Braunstein et al., 2017). Indeed, while the colloquial use of the term, “resilience” is important for frontline professionals, our findings suggest that emotion regulation is action defined when discussing the ability to be more resilient. As Rutter (2013) argues, research findings on resilience seldom translate into clear programmes of treatment or intervention. Our current findings provide conceptual implications that help contextualize the dynamic view of overcoming stress that Rutter proposes. Participants’ rich descriptions of resilience in-action provides perceptible accounts of emotion regulation defined by accounts of the abstract concept of resilience. It also suggests that, like lived mindfulness, learning may transfer in less conscious or direct ways. While resilience is a key component to positive change, our current findings suggest that further research on perceived resilience through emotion regulation strategies may prove fruitful in understanding mechanisms of change influenced by yoga-based programmes.

Findings surrounding self-care and self-compassion also have meaningful implications within current research. Increased self-care has been linked to positive outcomes in frontline professionals (Dyer et al., 2020). However, outcome-focused scopes do not provide information on how or why frontline professionals may engage with their own self-care activities. As noted, after participation in RISE, participants expressed a relief in having *permission* to engage with their self-care. This denotes a transformative quality, going from feelings of guilt surrounding self-care to commitment to spend time on self-care after RISE. Neff and Dahm (2017) propose that self-compassion increases acceptance of one’s emotions. In relation to participant perception, this implies that a successful programme for frontline professionals should encourage self-compassion, which may help overcome potential stigma and encourage self-care. This aspect seemed particularly pertinent in this population as recollections of romanticized beliefs around fast-paced work and selflessness seemed to influence the need for permission. Future research could establish baseline information on beliefs surrounding self-care and self-compassion, by way of *time for self* and *amount of negative self-talk*, which may provide pertinent information on the impact of yoga-based programmes on long-term change.

Our findings also suggest that yoga-based programmes for frontline professionals may benefit from facilitating group trainings that promote change by offering a shared experience with peers. Furthermore, efforts to empower the continued sharing of practices, constructs and experiences, and facilitating a change in their workplace culture may increase the lasting positive impact of a programme. Indeed, most findings provide underlying inference of grouping. Group, or peer, culture revolves around both explicit and implicit group interpretation of events, behaviours, and emotions (Macklem, 2008). Participants’ use of similar language suggests such an interpretation of events. As yoga practices encourage positive affect and mindfulness practices cultivate compassion, both of which have been shown to decrease avoidance of difficult emotions (Hildebrandt et al., 2019). Furthermore, the environment of learning in a retreat setting may provide a powerful space for transformation. The participants appeared to want to create the same environment in their home/work environment. Certainly, the social component of the yoga-based programme is probably most pronounced in the persistent theme sharing, yet there are few systematic methods of measuring the construct. The expressed excitement of sharing tools and facilitating a RISE experience outside of the retreat suggests a level of engagement in the RISE material that multiplies the positive impact of the programme beyond the individual. A more robust strategy of measuring sharing of RISE programme practices, constructs, and experiences after participation may provide more information on the potential mechanisms of long-term change.

While previous findings from the same participants as the current study support the quantitative evaluation of the RISE programme (Trent et al., 2018), it is important to note that the method of qualitative analysis is limited in scope. The nature of the interview and positionality of the interviewer impact the potential findings in such a way that causality cannot be inferred. Rather, the exploratory qualitative inquiry provides rich data that elicits more contextual and meaningful understanding of benefits and outcomes of participation in a yoga programme. Indeed, the qualitative inquiry was not designed to be explicitly evaluative or a programme assessment. Furthermore, the self-selection and relatively small population size does not offer a full scope of potential experiences of frontline professionals and is not meant to be generalized or transferred. Instead, our current findings present findings on participants' constructed understanding and inquiry directions for future research.

Our current findings further support RISE, an evidence-based practice designed for frontline professionals, by suggesting nuanced learning and skill acquisition through a variety of interconnected processes. For researchers interested in individuals exposed to stress and trauma, contextual understanding of increases in wellness outcomes (Trent et al., 2018) can provide meaningful tools for interventions and suggestions for assessment. For individuals in professions exposed to high amounts of stress and/or trauma, the current findings in conjunction with previous RISE reports (Dyer et al., 2020; Trent et al., 2018, 2019a) give support for the construction of positive experiences and evidence for mechanisms influencing outcomes in a yoga-based retreat setting.

Informed by this, further qualitative analysis could lead to a conceptual framework of change. In addition, inquiry around the phenomenological impact of tool use and programme setting as it relates to frontline professionals’ self-regulation skill acquisition and learning transfer may shed light on the adaptability and large-scale feasibility of such programmes for frontline professionals. Unique findings also suggest further research on the social mechanisms of resilience-based programmes like yoga. Future work, specifically in voluntary residential programmes that include social support and/or grouping, regarding resilience training, should consider inquiry methods that allow for further examination of the participants’ sharing of practices, constructs and experiences with others after the programme and the consequences of this.

An unconsidered component of both the yoga-based foundation in the RISE programme and the direction/content of the interviews was the spiritual dimension of their experience. Kwok et al. (2020) found that spiritual resilience was an important factor in coping and health-related quality of life. Additionally, spiritual orientation in nurses impacted the degree of compassion fatigue, burnout, and compassion satisfaction (Polat et al., 2020). Future work could consider if there is a perceived spiritual element to yoga-based programmes and how that might influence results.

An additional limitation in the current analysis was the lack of exploration on diversity in cases. While valence in experience was explored, the nature of the coding and interviews led to limited opportunities for comparison. Future work should explore the potential differences in experience.

The first aim of this project was to gain a rich contextual understanding of the impact of the RISE programme. Five main themes emerged (1) acquired behavioural skills and practices; (2) lived mindfulness; (3) resilience to stress and emotion regulation; (4) self-care and self-compassion; and (5) sharing with others the practices, constructs, and experiences after the programme. Further analysis of these themes demonstrates nuanced, integrated, and contextual meaning in relationship with reported transformation. Frontline professionals’ perceptions of benefits were experiential, this lived mindfulness offers an expanded depth of understanding and connection to the curriculum. In addition, the implied need for allowing self-care and potentially socializing self-compassion underlines the importance of self-care as a curricular component and shows the potential impact of participation in the RISE programme reaching beyond the individual. Furthermore, a social component was observed to have perceived importance. Participants reported with excitement sharing with others the tools learned and thereby spreading the benefits in their home environments. The second aim was to evaluate perceptions of the perceived long-term (3-months post-programme) impact of participation in RISE. Accounts of experience at Kripalu were positive, commonly citing the retreat experience as transformational. Upon leaving the retreat learning, or self-regulated skill acquisition, seemed to be both deliberately and organically transferred to frontline professionals’ daily lives.

Informed by these findings, suggestions for future research are directed towards analysing and measuring participant reported perceptions. In particular, understanding phenomenological components of lived mindfulness and perceived resilience through emotion regulation strategies to provide a more dynamic analysis of quantitative findings. In addition, obtaining a baseline measurement of time for self and negative self-talk to evaluate long-term change. Finally, we encourage establishing a strategy to measure participant sharing of programme practices, constructs, and experiences to better understand long-term impact.

## Supplementary Material

Supplemental Material

## Data Availability

Data sharing is not applicable to this article. Data generated for this paper was collected via audio files and verbatim transcriptions of confidential semi-structured interviews. It is not possible to share this data as conversational interviews contain inherent personal information.
