# Supplementary material for: “Making it okay”: professionals in high-stress environments construct their understanding of the impact of a yoga-based retreat designed to build resilience
Source: Int J Qual Stud Health Well-being. 2022 Feb 14;17(1):2025640. doi: 10.1080/17482631.2022.2025640 (PMC8925917; doi:10.1080/17482631.2022.2025640)
Supplement: Supplemental Material [file ZQHW_A_2025640_SM9247.docx]

**Appendix**

**Interview Guide**

The interview guide contained four main blocks of topics: (1) the practices, strategies, skills, concepts, insights that impacted them from the program, (2) any plans or intentions to incorporate yoga-based practices into their daily life, (3) whether their perspective and/or lifestyle and/or any aspect of their life changed as a result of the program, and (4) anything else they liked to share about the impact of the RISE program.

The questions for each topic were: (1) As you think back on your time at Kripalu Program, what still resonates with you today? (2) Did you either create a plan or set an intention to incorporate yoga-based practices, strategies, skills, concepts, and insights learned at Kripalu into your daily lifestyle? If so, please tell me about the plan or intention. Which of these did you intend to incorporate? How is this going? (3) Has your perspective and/or lifestyle and/or any aspect of your life changed as a result of experiencing the program at Kripalu? (4) As we wrap up is there anything else on your mind that you would like to share with me about the impact of the program you attended at Kripalu and/or the overall visit?
